# Antimicrobial resistance patterns of commensal *Escherichia coli* isolated from feces of non-diarrheic dogs in Grenada, West Indies

**DOI:** 10.14202/vetworld.2019.2070-2075

**Published:** 2019-12-27

**Authors:** Victor A. Amadi, Harry Hariharan, Ozioma A. Amadi, Vanessa Matthew-Belmar, Roxanne Nicholas-Thomas, Marta Lanza Perea, Kenrith Carter, Eugene Rennie, Keith Kalasi, Andy Alhassan, Richard M. Kabuusu, Grant Ugochukwu Alozie, Paul J. Fields, Rhonda Pinckney, Ravindra Sharma

**Affiliations:** 1 Department of Pathobiology, School of Veterinary Medicine, St. George’s University, St. George’s, Grenada, West Indies; 2 Department of Public Health and Preventative Medicine, School of Medicine, St. George’s University, Grenada, West Indies; 3 Department of Small Animal Medicine and Surgery, School of Veterinary Medicine, St. George’s University, St. George’s, Grenada, West Indies; 4 Department of Anatomy, Physiology and Pharmacology, School of Veterinary Medicine, St. George’s University, St. George’s, Grenada, West Indies; 5 Department of Small Animal Clinic, School of Veterinary Medicine, St. George’s University, St. George’s, Grenada, West Indies; 6 Department of Neuroscience, Physiology and Behavioral Science, School of Medicine, St. George’s University, St. George’s, Grenada, West Indies; 7 Office of Research, School of Graduate Studies, St. George’s University, St. George’s, Grenada, West Indies

**Keywords:** antimicrobial resistance, commensal *Escherichia coli*, dogs, Grenada

## Abstract

**Background and Aim:**

There is currently no published information on the prevalence and antimicrobial susceptibility patterns of commensal *Escherichia coli* in dogs of Grenada origin. Monitoring antimicrobial resistance helps in the empirical selection of antibiotics. This study determined the occurrence of *E. coli* including the O157:H7 serotype in feces of non-diarrheic dogs of Grenada origin and the antibiotic resistance pattern of the *E. coli* isolates.

**Materials and Methods:**

Fecal samples from 142 of the 144 (98.6%) dogs were culture positive for *E. coli*. Selection of up to three colonies from each of the 142 *E. coli*-positive samples yielded a total of 402 *E. coli* isolates, which were analyzed for the presence of non-sorbitol fermenting colonies, and O157-agglutination.

**Results:**

Of the 402 *E. coli* isolates, 30 (7.5%) were non-sorbitol fermenters. However, none of the 402 isolates gave a positive reaction (O157:H7) to the *E. coli* O157:H7 latex kit. Antimicrobial susceptibility tests against 12 antibiotics revealed low resistance rates to all the tested antibiotics except for tetracycline (Te) (23.4%), cephalothin (CF) (13.2%), and ampicillin (AM) (7.7%). Thirty-nine out of the 402 (9.7%), *E. coli* isolates were resistant to two or more antibiotics of different classes.

**Conclusion:**

This is the first report of isolation and antimicrobial susceptibilities of commensal *E. coli* from non-diarrheic dogs in Grenada. Some of the isolates (39/402 isolates, 9.7%) were resistant to multiple antibiotics. This study showed that presently, dogs in Grenada should not be considered a reservoir for the *E. coli* O157:H7 serotype and for multiple antibiotic-resistant *E. coli* strains. Among the 402 *E. coli* isolates, the resistance rate to drugs other than Te, CF, and AM was very low.

## Introduction

*Escherichia coli* is commonly found in the intestinal flora of humans and other mammals [1-3], including dogs. *E. coli* is excreted in feces and can be easily spread through food, water, and soil [1,2]. Most *E. coli* strains are non-pathogenic [4], however, some strains represent primary pathogens having the potential to cause diseases, especially diarrhea in humans and animals [5,6], suggesting zoonotic transmission [7].

Globally, more than 100 million cases of gastrointestinal illnesses and approximately 1 million deaths per year have been associated with pathogenic *E. coli* [8]. Shiga toxin-producing *E. coli* is a subdivision of the pathogenic *E. coli* group which represents an emerging group of zoonotic pathogens causing diarrhea, hemorrhagic colitis, and the life-threatening hemolytic uremic syndrome in humans [9]. Strains of O157 serogroup have been shown to be associated with many cases and outbreaks of human disease [10-13]. The intestines of animals are considered as a major reservoir and an ideal environment for the selection and transfer of antibiotic resistance genes. Studies have shown that *E. coli* can serve as reservoirs of antibiotic resistance genes [14] which have been efficiently transferred not only to other *E. coli* strains but also to other enteric pathogens of humans and animals [15]. There have been no published reports on the prevalence and antimicrobial susceptibility of *E. coli* including *E. coli* O157:H7 serotype in dogs in Grenada. However, several Grenada studies have shown that antibiotic resistance is minimal among different bacteria isolated from other animals, including wild and domestic animals, and crustaceans, and tetracycline (Te) resistance is common in Grenada [3,16-25].

The objectives of the present study were to determine the occurrence of *E. coli*, including the O157:H7 serotype in feces of non-diarrheic dogs of Grenada origin, and to study the antibiotic resistance pattern for the *E. coli* isolates.

## Materials and Methods

### Ethical approval

The St. George’s University Institutional Animal Care and Use Committee approved the study (IACUC 15006-R).

### Sample collection

The method used for the collection of samples has been previously described [17]. Briefly, the tested dogs were sampled from August to October 2016, the dog owners signed consent form to enter their dogs in the study, and the dogs were randomly selected from six of the seven parishes of the island of Grenada: St. George’s Parish 32, St. David’s Parish 28, St. Andrew’s Parish 26, St. Patrick’s Parish 24, St. Mark’s Parish 16, and St. John’s Parish 18 [17]. The gender, age, housing (indoor/outdoor or strictly indoor), breed, health history, history of antibiotic use, and date of sampling of the tested dogs were recorded [17]. Approximately 1-2 g of fresh fecal sample was collected from each dog [17,26], placed in a vial of transport medium and placed in a cooler with ice packs, and transported to the Bacteriology Laboratory, School of Veterinary Medicine, St. George’s University for laboratory analysis [17].

### Isolation and identification of *E. coli*

For the isolation of *E*. *coli*, the culture methods previously described [3,16,20] were used with slight modification. The contents of each vial were mixed and an aliquot (1 ml) was transferred into a tube containing 10 ml of tryptic soy broth (Remel, Lenexa, KS, USA) and incubated at 37°C for 18-24 h, as previously described [17]. After incubation, an aliquot was then subcultured onto MacConkey agar (MAC) (Remel, Lenexa, KS, USA) and incubated at 37°C for 24 h [3,16,20]. To increase the possibility of identifying *E. coli* O157:H7 in a sample, three pink to red color colonies with or without a zone of precipitated bile morphologically representing *E. coli* were subcultured onto individual MAC agar and incubated at 37°C for 24 h for isolation of pure colonies. The pure colonies were identification and confirmation of the colonies as *E. coli* using the analytical profile index strips (API20E-BioM6rieux, Hazelwood, MO) [3,16,20]. Atypical isolates identified as *E. coli* by API20E were also added in the study, as previously described [3,16,20].

### Identification of *E. coli* O157:H7 serotype

The identification of *E. coli* O157:H7 was performed using the methods, as previously described [3,16,20]. Briefly, the pure colonies were plated on sorbitol-MAC (Remel, Lenexa, KS, USA) and incubated at 37°C for 24 h. After incubation, the sorbitol-MAC plates were examined for the presence of non-sorbitol fermenting colonies. All the colonies (both the sorbitol and the non-sorbitol fermenting colonies) were then subjected to slide agglutination using *E. coli* O157:H7 latex kit – Remel Wellcolex × *E. coli* O157 rapid latex test (Remel Europe Ltd., Kent, UK). The *E. coli* isolates that showed negative reaction to the latex test kit were considered to be *E. coli* O157:H7 negative [3,16,20].

### Antimicrobial susceptibility test

Antimicrobial susceptibility testing was performed, as previously described [3,16,20] following the Clinical and Laboratory Standards Institute (CLSI) performance standards [27]. All the *E. coli* isolates were tested for susceptibility to the 12 antibiotics used in our previous study [17]. The antibiotic disks used were (BD BBL^™^ Sensi Disk^™^): Amoxicillin-clavulanic acid (AmC-20/10 µg), ampicillin (AM-10 µg), cefotaxime (CTX-30 µg), ceftazidime (CAZ-30 µg), cephalothin (CF-30 µg), chloramphenicol (C-30 µg), ciprofloxacin (CIP-5 µg), gentamicin (GM-10 µg), imipenem (IPM-10 µg), neomycin (N-30 µg), Te (Te-30 µg), and trimethoprim-sulfamethoxazole (SXT-1.25/23.75 µg) [17]. The inhibition zone sizes were interpreted based on CLSI guidelines [28] as recommended by the disk manufacturer (BD BBL^™^ Sensi Disk^™^). *E. coli* ATCC 25922 was used as a quality control strain [28].

### Statistical analysis

Online data analysis software: http://www.openepi.com/Menu/OE_Menu.htm was used for all the statistical analyses [17]. The OpenEpi – two by two table (Chi-squared analysis) was used to compare the differences in the proportions of female versus male dogs, indoor/outdoor dogs versus strictly indoor dogs, and <1 year versus >1 year dogs that were culture-positive for *E. coli* while the z-test analysis was used to compare the proportion of *E. coli* isolates showing single antibiotic resistance pattern versus those showing multiple antibiotic resistance pattern. The level of statistical significance was set at α=0.05. p<0.05 was considered statistically significant.

## Results

One hundred and forty-four non-diarrheic owned dogs were enrolled in the study. They comprised 140 (97%) indoor/outdoor dogs and four (3%) strictly indoor dogs. By gender, 56 (39%) were female and 88 (61%) were male, and by age, 46 (32%) were <1 year (<1), and 98 (68%) were >1 year (>1). All the tested dogs were mixed breed dogs known colloquially as pothounds. None of the dogs had a history of diarrhea or vomiting 3 weeks before sampling. Ten (7%) of the dogs had been treated with antibiotics 2 weeks before sampling; eight with doxycycline, one with cephalexin, and one with amoxicillin.

Of the 144 dogs tested, *E. coli* was isolated from the fecal samples of 142 (98.6%) dogs. The 142 positive dogs included 138 (98.6) of 140 indoor/outdoor dogs and all (100%) of four strictly indoor dogs, 55 (98.2%) of 56 female dogs and 87 (98.9%) of 88 male dogs, and 45 (97.8%) of 46 <1 year dogs and 97 (99%) of 98 >1 year dogs. There were no significant differences between the proportions of *E. coli*-positive female and male dogs (p=0.6849), indoor/outdoor and strictly indoor dogs (p=0.7875) or <1 year and >1 year dogs (p=0.8320). All of the dogs with a history of antibiotic treatment were *E. coli* positive.

Selection of up to three colonies with typical *E. coli* morphology from each of the 142 positive samples led to a total of 402 API20E-confirmed *E. coli* isolates: One from seven positive samples, two from 10 samples, and three from 125 positive samples. Of the 402 *E. coli* isolates, 30 (7.5%) were non-sorbitol fermenters. However, none of the 402 isolates gave a positive reaction (O157:H7) to the *E. coli* O157:H7 latex kit.

Based on the results of antimicrobial susceptibility testing by the Kirby-Bauer assay, all the 402 *E. coli* isolates were susceptible to IPM, 400 (99.5%) isolates were susceptible to CTX and CAZ. Two isolates were resistant to CAZ. These two CAZ resistant isolates were also intermediate resistant to CF. The most prevalent resistance rate observed was for Te (23.4%) followed by CF (13.2%) (Table-1). Ten of the tested dogs had received antibiotics 2 weeks before sampling. Antimicrobial-resistant *E. coli* strains were isolated from seven out of the ten dogs. The most common resistance seen among the dogs was to Te, of which isolated from six of the seven dogs showed resistance to Te. Some of the *E. coli* isolates (39/402 isolates, 9.7%) were resistant to multiple antibiotics (multidrug resistance [MDR] to two or more antibiotics of different classes) (Table-2), while some (113/402 isolates, 28.1%) showed resistance to a single antibiotic. The isolates showing MDR were significantly fewer than those showing resistance to one antibiotic (p<0.001). The most common MDR pattern observed was to three antibiotics (AM/SXT/Te), of which eight *E. coli* isolates showed the pattern. Six isolates showed MDR to four antibiotics (AM/C/SXT/Te) and two other isolates also showed MDR to four antibiotics (AM/CF/SXT/Te) (Table-2).

**Table-1:** Antibiotic susceptibility profiles of 402 *Escherichia coli* isolated from feces of non-diarrheic owned dogs in Grenada.

Antibiotic (disk concentration [μg])	Resistant n (%)*	Intermediate n (%)*	Susceptible n (%)*
Amoxicillin-clavulanic acid (30)	2 (0.5)	8 (2)	392 (97.5)
Ampicillin (10)	31 (7.7)	14 (3.5)	357 (88.8)
Cefotaxime (30)	0	2 (0.5)	400 (99.5)
Ceftazidime (30)	2 (0.5)	0	400 (99.5)
Cephalothin (30)	53 (13.2)	197 (49)	152 (37.8)
Chloramphenicol (30)	6 (1.5)	1 (0.2)	395 (98.3)
Ciprofloxacin (5)	3 (0.7)	0	399 (99.3)
Gentamicin (10)	6 (1.5)	2 (0.5)	394 (98)
Imipenem (10)	0	0	402 (100)
Neomycin (30)	3 (0.7)	95 (23.6)	304 (75.6)
Tetracycline (30)	94 (23.4)	13 (3.2)	295 (73.4)
Trimethoprim-sulfamethoxazole (1.25, 23.75)	20 (5)	1 (0.2)	381 (94.8)

* #=Number, % (percentage)=Values are rounded up and down to one decimal place

**Table-2:** MDR patterns of the 39 *Escherichia coli* isolates from feces of non-diarrheic owned dogs in Grenada.

Multiple antibiotic resistance pattern	Number of antibiotics to which the isolates are resistant	Number of isolates showing MDR (%)
GM/Te	2	5 (1.2)
N/Te	2	2 (0.5)
AM/Te	2	7 (1.7)
SXT/Te	2	1 (0.25)
CF/Te	2	3 (0.75)
CF/AM	2	1 (0.25)
AmC/CF	2	1 (0.25)
AM/GM/Te	3	1 (0.25)
AM/SXT/Te	3	8 (2)
N/SXT/Te	3	1 (0.25)
AM/CF/Te	3	1 (0.25)
AM/C/SXT/Te	4	6 (1.5)
AM/CF/SXT/Te	4	2 (0.5)

% (percentage)=Values are rounded up and down to one decimal place. AmC=Amoxicillin-clavulanic acid, AM=Ampicillin, CTX=Cefotaxime, CAZ=Ceftazidime, CF=Cephalothin, C=Chloramphenicol, CIP=Ciprofloxacin, GM=Gentamicin, IPM=Imipenem, N=Neomycin, Te=Tetracycline, SXT=Trimethoprim-sulfamethoxazole, MDR=Multidrug resistance (resistance to two or more antibiotics of different classes)

## Discussion

In this study, *E. coli* was isolated from 142/144 (98.6%) dogs. The results of this study indicate that presently, non-diarrheic dogs in Grenada harbor *E. coli* in their gastrointestinal tracts and the occurrence is widespread among dogs in Grenada irrespective of their distribution, gender, housing, breed, health history, and history of antibiotic use. It is possible that the two culture-negative dogs in this study may still be sub-clinical shedders of *E. coli* because fecal shedding can be dynamic.

Our study showed a high prevalence rate of *E. coli* in non-diarrheic dogs in Grenada, none of the tested dogs were positive for *E. coli* O157:H7 serotype based on our agglutination test results. In Japan, a 3-year (1996-1998) epidemiological surveillance of *E. coli* O157:H7 in dogs and cats showed that only one dog kept by a human patient infected with *E. coli* O157:H7 tested positive for *E. coli* O157:H7 [29]. The authors reported that companion animals may not give harbor to *E. coli* O157:H7 [29]. In the present study, 30 (7.5%) of the *E. coli* isolates were non-sorbitol fermenters that gave a negative reaction (no O157-agglutinating) to the *E. coli* O157:H7 latex kits. This study was designed to target only the *E. coli* O157:H7 serotype, which is usually non-sorbitol fermenters that give a positive reaction to the *E. coli* O157:H7 latex kits. Hence, the 30 *E. coli* isolates that were non-sorbitol fermenters that gave a negative reaction to the *E. coli* O157:H7 latex kits were not identified in relation to their serotypes.

Companion animals represent potential sources of spread of antimicrobial resistance due to the extensive use of antimicrobial agents in these animals and their close contact with humans [30]. Commensal *E. coli* in the intestines of animals, including dogs, can develop antibiotic resistance on exposure to antimicrobial agents. Considering the close contact that humans have with dogs, the high levels of antibiotic-resistant *E. coli* in canine feces may be a potential source of antibiotic-resistant bacteria or resistance determinants [31,32]. In the present study, the most prevalent resistance rate observed was for Te (23.4%) followed by CF (13.2%). In this study, 10 (7%) of the tested dogs had received antibiotics (eight received doxycycline, one received cephalexin, and one amoxicillin) 2 weeks before sampling. The most common resistance seen among these dogs was resistance to Te. It is important to note that the household use of antibiotics by individuals in the homes where the dogs lived were not determined in this study. Antimicrobial agents are used therapeutically in humans and animals for control of bacterial infections [33]. It is possible that the resistant *E. coli* may have been acquired by the dogs through direct exposure to the individuals in the homes where they lived. Further study is needed to elucidate the role of individuals (in the homes where the dogs lived) in the transmission of the resistant *E. coli* strains. In Grenada, Te resistance is high, several studies have shown that Te resistance is common among a variety of Gram-negative bacteria from different sources [3,16-25]. It is noteworthy that chlortetracycline is routinely used as a feed additive for food animals such as pigs in Grenada. Furthermore, oxytetracycline is used for the treatment of pigs for bacterial infections [1]. The food animals can easily contaminate human dwellings, fields, vegetables, and water sources with their feces. This, along with the environmental robustness of *E. coli* and large dog populations, may lead to long-term human and animal exposure to Te-resistant bacteria. In this study, two isolates were resistant to CAZ. These two CAZ-resistant isolates were also intermediate resistant to CF. It is noteworthy that the two CAZ-resistant isolates may be producing the extended-spectrum beta-lactamases or AmpC β-lactamases, although the current study was not designed to determine the resistance mechanisms involved.

In the present study, 9.7% of isolates showed MDR. In a recent study in the UK, 13.1% of fecal samples from hospitalized companion animals, 93% being dogs, yielded MDR *E. coli* [34]. In other studies, 15% of community-based dogs in the UK [35] and 32% of stray dogs in Korea [36] were positive for MDR. Resistance can develop by several mechanisms. Resistance to Te was the highest (23%) among all antibiotics used in this study, and it was characterized by MDR in 95% of 39 isolates in this study. Beta-lactam resistance was seen in 77% of MDR isolates in this study *. E. coli* is the predominant bacterial species associated with urinary tract infection (UTI) in dogs. MDR *E. coli* can be a problem in cases of UTI in dogs [37]. Emerging resistance to extended-spectrum cephalosporins including CAZ is of concern. *E. coli* strains from pet dogs and owners have been shown to have similar resistance patterns and genetic relatedness [38].

## Conclusion

We documented the prevalence of *E. coli* in the feces of non-diarrheic owned dogs in Grenada to be 98.6%. This current study showed that presently, dogs in Grenada should not be considered reservoirs of *E. coli* O157:H7 serotype. Among the commensal isolates, resistance rates to drugs other than Te, CF, and AM were very low. Resistance to two or more antibiotics was seen only in 9.7% of the *E. coli* isolates. This indicates that non-diarrheic dogs in Grenada are presently not main reservoirs for multiple resistant *E. coli* strains.

## Authors’ Contributions

VAA and HH designed the protocol and were involved with sample collection. MLP, KC, ER, KK, and GUA were involved with sample collection. VAA, VM, RN, KK, and AA carried out all the laboratory work. VAA, RMK, and PJF performed data analysis and supervised the study. VAA wrote the paper, and OAA, HH, RP, RS were involved in the drafting of the manuscript. All authors revised, read, and approved the final manuscript.
